# CorrQuant: Development of a Web Platform for Image-Based Corrosion Quantification

**DOI:** 10.3390/jimaging12080359

**Published:** 2026-08-06

**Authors:** Cynthia Martínez-Ramos, Citlalli Gaona-Tiburcio, Erick Maldonado-Bandala, Demetrio Nieves-Mendoza, Laura Landa-Ruíz, Maria Lara-Banda, Francisco Estupinan-Lopez, Miguel Angel Baltazar-Zamora, Jesús Manuel Jáquez-Muñoz, Jose Cabral-Miramontes, Facundo Almeraya-Calderón

**Affiliations:** 1 Universidad Autónoma de Nuevo León. FIME, Centro de Investigación e Innovación en Ingeniería Aeronáutica (CIIIA), San Nicolás de Los Garza 66455, Mexico; maria.laraba@uanl.edu.mx (M.L.-B.); francisco.estupinanlp@uanl.edu.mx (F.E.-L.); jose.cabralmr@uanl.edu.mx (J.C.-M.); facundo.almerayacld@uanl.edu.mx (F.A.-C.); 2 Facultad de Ingeniería Civil, Universidad Veracruzana, Xalapa 91000, Mexico; erimaldonado@uv.mx (E.M.-B.); dnieves@uv.mx (D.N.-M.); lalanda@uv.mx (L.L.-R.); mbaltazar@uv.mx (M.A.B.-Z.); 3 Metal-Mechanics Department, Instituto Tecnológico de Ciudad Juárez, Av. Tecnológico 1340, Ciudad Juárez 32500, Mexico; jesus.jm01@cdjuarez.tecnm.mx

**Keywords:** corrosion quantification, digital image processing, computer vision, image segmentation

## Abstract

Corrosion remains one of the principal causes of degradation in metallic structures across a wide range of industrial sectors. Although visual inspection is routinely employed for preliminary corrosion assessment, its effectiveness depends heavily on operator experience and subjective interpretation. This work introduces CorrQuant, a web-based computer vision platform designed to transform qualitative corrosion images into quantitative measurements of corrosion extent and morphology. The proposed methodology processes images acquired with conventional mobile devices and integrates geometric calibration using a reference coin, perspective correction, adaptive image enhancement through Contrast Limited Adaptive Histogram Equalization (CLAHE), multi-descriptor feature extraction, and consensus-based corrosion segmentation. The detected corrosion regions are subsequently quantified to determine corrosion area, surface coverage, spatial distribution, morphological descriptors, and corrosion intensity maps. The methodology was verified using an aluminum specimen with a known corrosion area of 143 mm^2^ under both controlled illumination and optical stress-test conditions. Under standard acquisition conditions, corrosion-area estimation accuracies exceeding 90% were achieved. Additional evaluations under red illumination, fisheye, blur, and kaleidoscope distortions demonstrated that the proposed framework is considerably more sensitive to degradation of local image information than to variations in illumination spectrum. These results demonstrate the robustness of the proposed multi-descriptor voting strategy while defining the operational limits of the platform under challenging image acquisition conditions.

## 1. Introduction

Corrosion is one of the principal causes of degradation in metallic structures and engineering infrastructure, affecting transportation systems, industrial facilities, pipelines, and numerous other metallic assets [1]. If left undetected, corrosion progressively compromises structural integrity through complex electrochemical processes governed by environmental thermodynamics and reaction kinetics [2]. Under aggressive service conditions, material degradation may accelerate considerably, leading to significant metal loss, reduced structural reliability, increased maintenance costs, and, ultimately, component failure.

Among the various forms of corrosion, localized corrosion is particularly critical because it often initiates at the microscopic scale and may remain undetected until substantial damage has already occurred. Pitting, crevice, intergranular, and transgranular corrosion are representative localized degradation mechanisms that, despite their distinct characteristics, share the ability to produce highly concentrated damage within limited surface regions, frequently escaping detection during routine visual inspections [3,4,5].

Computer vision applications within corrosion research are not a new concept in and of themselves; many researchers have sought to connect the processing power and techniques found in image processing to advance and improve on the identification and classification of corrosion. One particular challenge of this endeavor lies in its interdisciplinary nature, requiring the integration of programming, data science, statistics, mathematics, and corrosion science to develop reliable methodologies. Consequently, the evolution of computer vision within corrosion research has been characterized by the gradual incorporation of increasingly sophisticated image-analysis techniques. Despite these advances, most reported methodologies have focused on specific image-processing tasks or laboratory-oriented applications, leaving a need for integrated and accessible platforms capable of performing quantitative corrosion assessment from conventional digital images.

More than four decades ago, Baer et al. in 1985 [6] anticipated that advanced surface-analysis techniques would eventually become part of the standard toolkit used in corrosion research. Although this prediction originally referred to analytical methods such as Auger Electron Spectroscopy (AES), X-ray Photoelectron Spectroscopy (XPS), Rutherford Backscattering Spectrometry (RBS), Nuclear Reaction Analysis (NRA), and Raman spectroscopy, subsequent advances in digital imaging, computational power, and portable imaging devices have greatly expanded the range of techniques available for surface characterization.

Quantitative image analysis based on stereological principles has become an established approach for obtaining objective measurements from micrographs. Stereology provides statistically rigorous methods for estimating area fractions, feature distributions, and other microstructural parameters from two-dimensional images, thereby reducing the subjectivity associated with conventional visual inspection [7]. These concepts have subsequently been applied in corrosion research to enable objective characterization of corrosion damage.

As digital computing became increasingly available, researchers began exploring ways of extracting quantitative information directly from images. K. Komai and M. Noguchi (1986) used stereo SEM photographs to reconstruct the three-dimensional morphology of corrosion-fatigue fracture surfaces [8]. The method relied on stereoscopy, in which images of the same surface are acquired at different tilt angles. To determine surface height, corresponding points between the stereo image pair were identified using the Sequential Similarity Detection Algorithm (SSDA) and cross-correlation methods. The displacement between matching regions was then converted into height information, enabling the generation of three-dimensional topographic maps and contour plots of the fracture surface. This work also demonstrated that image-processing techniques could support the quantitative evaluation of coupled degradation mechanisms, such as corrosion-fatigue, thereby broadening the scope of computer vision applications in materials engineering.

A further milestone was achieved by Quinn et al. [9], who introduced three-dimensional reconstruction of corroded surfaces using one of the earliest television-based digital image analysis systems. Their methodology enabled quantitative characterization of pit morphology, surface roughness, and topographical features that could not be adequately described using conventional two-dimensional images alone. By incorporating three-dimensional surface reconstruction, this work significantly expanded the capability of image analysis to evaluate the geometry and severity of corrosion damage, establishing an important foundation for subsequent surface-characterization techniques. Despite these advances in quantitative surface characterization, image-analysis methodologies remained largely confined to specialized laboratory environments and required substantial user expertise, limiting their broader adoption in routine corrosion assessment.

At this stage, image-analysis systems have become more accessible, and researchers have explored new ways of incorporating them into corrosion analysis and producing unprecedented techniques for the quantification of corrosion, expanding the possibilities of what could be achieved through image-based measurements; however, the techniques remained strongly tied to laboratory equipment and controlled experimental conditions.

Entering the aughts, the work of K.Y. Choi and S.S. Kim [10] introduced a morphology-based approach for corrosion damage classification through digital image processing. The authors characterized corrosion using color, texture, and shape descriptors extracted from digital images. RGB images were transformed into the HSI color space for color analysis; texture features were quantified using Gray-Level Co-occurrence Matrices (GLCM); and morphological parameters such as area, perimeter, shape factor, and axis ratios were extracted from corrosion defects. These descriptors were combined into an 18-dimensional feature vector and analyzed through multidimensional scaling procedures to classify five different forms of corrosion damage.

Of a similar nature, the work of E.N. Codaro et al. [11] developed an image-processing methodology for pitting corrosion evaluation based on quantitative morphology analysis. Microscopy images were segmented through thresholding, hole-filling, binary morphological operations, and watershed filtering to isolate individual pits. Geometric descriptors, including pit area, perimeter, depth, width, aspect ratio, and a proposed Area-Box (AB) factor, were then extracted and used to classify pit geometries as conical, hemispherical, or cylindrical, while providing quantitative measures of pit morphology and severity.

Atmospheric corrosion is also addressed through image analysis by L. Tao et al. [12] whose research took a different approach to image processing; rather than segmenting or classifying, they centered around the image as a texture signal from which to extract information. Corrosion images were denoised and subjected to three-level wavelet transformation to obtain the sub-images corresponding to different spatial frequencies and orientations. The energy of each sub-image was calculated and combined into an image-energy loss parameter. When compared with conventional weight-loss measurements, the image-based analysis produced consistent rankings of alloy corrosion performance, suggesting that image-energy metrics could serve as a quantitative indicator of corrosion damage.

A perusal of the most recent years reveals a substantial surge in image-based corrosion research. One such example is the work of M. Khayatazad et al. [13], who developed an automated corrosion-detection methodology for steel structures by combining texture analysis and color-based recognition to identify and map corroded regions in digital images. Pursuing onto the torrential growth of multidisciplinary corrosion research, Forkan et al. [14] proposed CorrDetector which constitutes an ensemble deep-learning framework with convolutional neural networks for corrosion detection in drone-acquired images of industrial sites and structures, whose approach solves the ever-pressing issue regarding analysis of difficult-to-access areas while reducing the reliance on manual visual assessment.

Particularly relevant to the present work is the research of Moreira et al. [15], who employed the freely available ImageJ software to quantify corrosion damage in specimens subjected to salt spray testing; this showcases that quantitative corrosion measurements could be obtained using widely available image-analysis software and standard digital photographs.

Ramírez Barat et al. [16] presented the MIPAC project (Monitoring by Image Processing and Citizen Science for the Conservation of Cultural Heritage Materials) preliminary results, which explored corrosion detection through color changes extracted from crowdsourced smartphone photographs. Images acquired under real-world conditions were uploaded through a dedicated mobile application to remote processing servers, where image calibration and colorimetric analysis were performed. The extracted color information was then correlated with conventional corrosion measurements, demonstrating the potential of accessible image-based monitoring using non-specialized devices.

More recently, the integration of photogrammetry and artificial intelligence has enabled quantitative corrosion assessment at infrastructure scale. P. Savino et al. [17] proposed a methodology for automated corrosion quantification in steel transmission towers by combining UAV photogrammetry and deep-learning semantic segmentation. Corroded regions were identified using a DeepLabv3+ convolutional neural network and projected onto photogrammetric orthomosaics, allowing corrosion extent to be measured with metrical accuracy.

Despite the significant advances achieved in image-based corrosion assessment, several challenges remain. Most existing methodologies are focused on specific image-processing algorithms or laboratory-oriented implementations, often requiring specialized software, considerable user intervention, or advanced programming expertise. Consequently, the development of accessible, integrated, and reproducible platforms capable of performing quantitative corrosion assessment from conventional digital images remains limited.

To address these limitations, this work introduces CorrQuant, a web-based platform specifically designed for automated image-based corrosion quantification. The platform integrates image calibration, preprocessing, adaptive segmentation, multi-descriptor analysis, and quantitative corrosion measurement into a unified workflow that can be accessed through a standard web interface. Unlike conventional image-analysis software that requires extensive manual interaction, CorrQuant was developed to provide an intuitive, reproducible, and objective framework for corrosion assessment while maintaining flexibility under different image acquisition conditions.

The proposed methodology was evaluated using representative corrosion datasets acquired under controlled and challenging imaging conditions. Its performance was assessed through quantitative segmentation metrics and comparative analyses, demonstrating the robustness of the multi-descriptor voting strategy and the applicability of the platform for reliable corrosion quantification.

## 2. Materials and Methods

### 2.1. System Development Environment

CorrQuant is a web-based platform developed for automated image-based corrosion quantification. The application was implemented using a client–server architecture in which the graphical user interface (frontend) communicates with the computational image-processing modules (backend). The frontend was developed using HyperText Markup Language (HTML), Cascading Style Sheets (CSS), and JavaScript, whereas the backend was implemented in Python 3.14.6 using the Flask web framework [18], which manages communication between the user interface and the processing pipeline. This architecture enables image upload, parameter transmission, computational processing, and visualization of quantitative results directly through a standard web browser.

CorrQuant follows a client–server architecture composed of a frontend and backend subsystem. The frontend provides the graphical user interface through which users upload images, perform calibration, define regions of interest, and visualize results. The backend executes all computational operations, including image correction, feature extraction, corrosion detection, quantitative analysis, and result generation.

For deployment, the application was hosted on a cloud-based server, allowing remote access without requiring local installation of the analysis software. Development and validation of the processing pipeline were performed on a workstation equipped with an AMD Ryzen™ 3 PRO 4350G processor with Radeon™ Graphics (4 physical cores and 8 logical processors; Advanced Micro Devices (AMD), Santa Clara, CA, USA). The application was developed using Python 3.14.6 in Visual Studio Code version 1.104 (Microsoft Corporation, Redmond, WA, USA). All testing was performed on the same workstation to ensure reproducibility of the image-processing pipeline.

### 2.2. The Frontend Interface

Design of the interface is based on accessibility and intuitive commands with minimal user involvement [19]. The webpage includes instructional content explaining the methodology implemented by CorrQuant and providing guidance for interpreting the generated results.

After an image has been successfully uploaded, the analysis controls initiate the calibration stage; during this process, the user selects a reference coin type from the dropdown menu to establish a physical scale conversion between image pixels and real-world dimensions (px/mm). The reference coin in the image provided to the system is then encompassed by the user using either a circular overlay or elliptical for cases in which image perspective distortion is present. Once calibration parameters have been confirmed by the user, the corresponding scale information is displayed, and the workflow proceeds to the next stage.

The subsequent page involves the region-of-interest (ROI) selection; users are instructed to define the specific area to be analyzed through polygonal selection of a minimum of three points. This step ensures that all background information and other possible outside interferences such as texture, shadows or glares are excluded, surrounding only relevant surface information. After ROI confirmation the image-processing pipeline is executed, and the calculated corrosion metrics are returned to the interface.

Finally, the Section 3 presents both quantitative measurements and visual representations of the detected corrosion. Quantitative indicators include the corroded area, corrosion coverage percentage, number of detected regions, total ROI area, largest region area, largest region share percentage, and boundary complexity. Visual outputs include a corrosion intensity map, in which warmer colors indicate areas with stronger corrosion signatures, and a corrosion overlay superimposed on the original specimen image.

### 2.3. Backend Processing Pipeline

The backend architecture of CorrQuant is organized into nine processing modules and a central execution module that coordinates the workflow throughout the system. The execution module receives user inputs from the frontend, manages the sequential execution of the image-processing and analysis stages, and returns the generated results for visualization. This centralized design ensures a consistent processing sequence, facilitates data exchange among modules, and enhances the modularity and maintainability of the platform.

#### 2.3.1. Perspective Correction

The first stage of the processing pipeline consists of a perspective correction procedure applied only when the user selects an elliptical calibration object during the calibration stage; An elliptical appearance of the reference coin indicates geometric compression caused by image acquisition at an oblique viewing angle [20]. If the calibration object is selected as a circle, no correction is applied and the image proceeds directly to the subsequent processing stages.

For elliptical calibrations, the major and minor radii of the selected ellipse are obtained and used to calculate a compression ratio representing the degree of geometric deformation. The shorter axis of the ellipse is then scaled by this ratio through an affine transformation [20,21], restoring the elliptical projection to an approximately circular geometry. Translation terms are incorporated into the transformation matrix to preserve the position of the coin center during scaling. The affine transformation is applied to the entire image using bicubic interpolation, producing a corrected image that compensates for perspective-induced compression while maintaining the spatial relationships between image features [21]. In addition to the corrected image, the module returns the compression ratio and scaling factors used during the correction process for subsequent calculations.

#### 2.3.2. Image Correction

The next executed stage corresponds to a subtle generalized image enhancement applied to the input image to improve the visibility of corrosion features and reduce the influence of illumination inconsistencies. First, the image is transformed into the CIELAB color space to separate luminance from chromatic information and allow for brightness enhancement to be performed independently from the color characteristics.

The Luminance (L) channel obtained from the transformed is enhanced through Contrast Limited Adaptive Histogram Equalization (CLAHE) which equalizes and reduces contrast while preventing the over-amplification of noise [22,23]. In the present implementation, CLAHE was applied using a clip limit of 2.0 and a tile grid size of 8 × 8. After the enhancement the modified luminance channel is recombined with the original chromatic channels and transformed back into its original color representation. A bilateral filter is subsequently applied to the enhanced image in order to reduce small-scale intensity variations while preserving significant edges and boundaries [24]. The bilateral filter was implemented using a kernel diameter of 5 pixels with color and spatial sigma values of 50.

#### 2.3.3. Feature Extraction

Corrosion does not manifest through a single visual characteristic; thus, multiple complementary descriptors were employed to capture its different manifestations such as simultaneous change in surface coloration, texture, roughness, and boundary definition [10,11,13,15]. This particular stage acts as a collection step for the visual descriptors that will be employed in the following processing stages. The chromatic a and b channels from the enhanced image are first extracted which provide information regarding color variations that may be associated with corrosion products and surface discoloration. Additional texture descriptors are then generated, including a variance map, Laplacian map, edge map, and entropy map.

The variance map is obtained through local neighborhood statistics and indicates regions exhibiting elevated intensity variability, which can be indicative of surface heterogeneity associated with corrosion. The Laplacian map identifies abrupt intensity transitions and localized structural changes, while the edge map highlights boundaries and discontinuities present on the specimen surface. The final feature of interest consists of the entropy map, which quantifies local texture complexity and randomness, characteristics commonly observed in corroded regions [21,25].

To ensure that subsequent analyses are restricted to the specimen under evaluation, all generated feature maps are masked according to the user-defined region of interest before being forwarded to the candidate region generation stage.

#### 2.3.4. Candidate Region Generation

With the previously extracted features a consensus was generated through an adaptive voting system. To effectuate this, each feature map was independently evaluated and transformed into a binary vote indicating whether a given pixel deviated significantly from the normal behavior of the specimen.

Adaptive thresholds were calculated from the statistical properties of the region of interest. For the variance, Laplacian, and entropy maps, thresholds were defined as the sum of the mean feature response and one standard deviation (Equation (1)):

(1) $$T_{i}=\mu_{i}+\sigma_{i}$$

and the pixels exceeding these thresholds were assigned a vote. Similarly, pixels exhibiting significant chromatic deviations from the average specimen coloration within the LAB color space were assigned a vote, while edge information was incorporated through a single dilation operation using a 3 × 3 structuring element prior to voting to reinforce potential corrosion boundaries.

The individual votes were then accumulated into a vote map representing the degree of agreement among the descriptors, for each pixel (x,y) the vote score was calculated (Equation (2)) as:

(2) $$V\left(x,y\right)=V_{B}\left(x,y\right)+V_{Var}\left(x,y\right)+V_{Lap}\left(x,y\right)+V_{Ent}\left(x,y\right)+V_{Edge}\left(x,y\right)$$

where each descriptor contributes a binary vote with a value of 1 when its voting criterion is satisfied and 0 otherwise. A pixel was classified as a candidate corrosion pixel only when at least two independent features identified it as anomalous (Equation (3)):

(3) $$C\left(x,y\right)=\left\{\begin{array}{cc} 1, & V\left(x,y\right)\geq 2\\ 0, & V\left(x,y\right)<2 \end{array}\right.$$

where $C\left(x,y\right)$ represents the candidate region mask, which was subsequently refined through morphological opening and closing using a 5 × 5 structuring element to remove isolated artifacts and improve region continuity [21].

As a final refinement stage, connected candidate regions were analyzed individually. Only connected regions containing at least 50 pixels were considered for the subsequent region-growing stage. For each eligible region, the mean LAB b-channel value was calculated, and neighboring pixels were incorporated when their LAB b intensity differed by less than six LAB b-channel intensity values from the regional mean. Candidate expansion was performed by first dilating each region using a 5 × 5 structuring element and subsequently evaluating only neighboring pixels within the region of interest satisfying the chromatic similarity criterion.

#### 2.3.5. Region Extraction

In this stage, the detected pixels are transformed into individual corrosion regions, and their geometric properties are calculated. First, the binary candidate mask $C\left(x,y\right)$ is scanned and pixels belonging to the same connected structure were grouped into individual regions using an 8-neighbor connectivity criterion [21].

For each detected region geometric descriptors including area, centroid location, and perimeter are calculated. Region perimeters are obtained through contour extraction and arc-length measurements [21] which provide information regarding the spatial extent and boundary characteristics of each corrosion feature. The connected-component analysis additionally provides positional information for each region, allowing individual corrosion sites to be localized within the specimen.

The extracted descriptors are subsequently stored and combined into a unified region mask representing all detected corrosion regions within the region of interest. This region mask serves as the basis for the geometric, distributional, and morphological analyses performed in the following stages of the processing pipeline.

#### 2.3.6. Distribution Analysis

Up till this stage, the system is no longer trying to locate corrosion but rather defining how corrosion is distributed across the surface by characterizing the spatial organization and relative size distribution of the detected corrosion regions.

The dominance index was defined (Equation (4)) as:

(4) $$D=\frac{A_{max}}{\sum_{i=1}^{n}A_{i}}$$

where $A_{max}$ represents the area of the largest detected region and $\sum_{i=1}^{n}A_{i}$ corresponds to the total corroded area. This metric describes the extent to which the corrosion pattern is governed by a single dominant region. Values approaching unity indicate that corrosion is concentrated within a single large feature, whereas lower values suggest a more distributed corrosion pattern.

The percentage of the region of interest affected by corrosion was quantified through the coverage percentage (Equation (5)):

(5) $$C=100\left(\frac{P_{c}}{P_{ROI}}\right)$$

where P_C_ corresponds to the number of pixels classified as corrosion and P_ROI_ represents the total number of pixels contained within the region of interest, providing a direct measure of the proportion of the inspected surface occupied by detected corrosion.

#### 2.3.7. Morphology Analysis

The extracted corrosion regions were evaluated to characterize their size variability and boundary complexity in order to define the regularity or irregularity of the detected corrosion features.

For each extracted region, a compactness descriptor was calculated according (Equation (6)) to:

(6) $$C_{i}=\frac{P_{i}^{2}}{A_{i}}$$

where P_i_ and A_i_ represent the perimeter and area of region (i), respectively. This descriptor provides a measure of boundary irregularity, with larger values corresponding to increasingly complex region geometries.

The compactness values obtained from all detected regions were summarized through percentile statistics from which a complexity index was calculated (Equation (7)) as:

(7) $$CI=\frac{CP_{95}}{CP_{50}}$$

where CP_95_ and CP_50_ correspond to the 95th and 50th percentile compactness values, respectively. The resulting complexity index provides a quantitative measure of the presence of highly irregular corrosion morphologies relative to the typical region shape and is reported as one of the primary outputs of the analysis.

#### 2.3.8. Geometric Quantification

The final corrosion regions were converted from image coordinates into physical measurements using the calibration factor obtained during the calibration stage. This conversion allows corrosion extent to be reported in real-world units rather than pixel counts.

The total area contained within the user-defined region of interest was first determined according (Equation (8)) to:

(8) $$\left[A_{ROI}=\frac{P_{ROI}}{{\left(px/mm\right)}^{2}}\right]$$

where (P_ROI_) corresponds to the number of pixels contained within the region of interest and (px/mm) is the pixel-to-millimeter calibration factor.

The total corroded area was subsequently calculated (Equation (9)) as:

(9) $$\left[A_{C}=\frac{P_{C}}{{\left(px/mm\right)}^{2}}\right]$$

where (P_C_) represents the number of pixels classified as corrosion. This metric is reported as the corroded area in square millimeters.

The area of the largest detected corrosion region was similarly converted into physical units according (Equation (10)) to:

(10) $$\left[A_{max}=\frac{P_{max}}{{\left(px/mm\right)}^{2}}\right]$$

where (P_max_) corresponds to the pixel area of the largest detected corrosion region. This descriptor provides an indication of the size of the most dominant corrosion feature present within the specimen.

To characterize the spatial concentration of corrosion features, the region density was calculated (Equation (11)) as:

(11) $$\left[RD=\frac{N}{A_{ROI,cm^{2}}}\right]$$

where (N) is the number of detected corrosion regions and (A_ROI,cm2_) is the area of the region of interest expressed in square centimeters. The resulting metric represents the number of corrosion regions per unit surface area and is reported as regions per square centimeter.

#### 2.3.9. Corrosion Intensity Mapping

To facilitate visual interpretation of the analysis results, a corrosion intensity map was generated from the vote map obtained during candidate region generation. Since the vote map represents the degree of agreement among the extracted feature descriptors, regions receiving a larger number of votes correspond to locations exhibiting stronger corrosion signatures.

The vote map was first normalized to a standardized intensity range and subsequently transformed into a color-coded heatmap. A Turbo colormap was employed to enhance visual contrast [26], where cooler colors indicate lower voting consensus and warmer colors indicate higher agreement among the feature descriptors [27,28,29].

Finally, the heatmap was masked to the user-defined region of interest and superimposed onto the original specimen image, producing a corrosion overlay that allows the spatial distribution and relative strength of the detected corrosion signatures to be visualized alongside the original surface condition.

### 2.4. Dataset Specimens

To evaluate the performance of the proposed methodology a three-component verification strategy was implemented composed by: (A) controlled evaluation under standardized conditions, (B) multi-specimen analysis, and (C) ground-truth synthetic corrosion evaluation.

**Figure 1:**
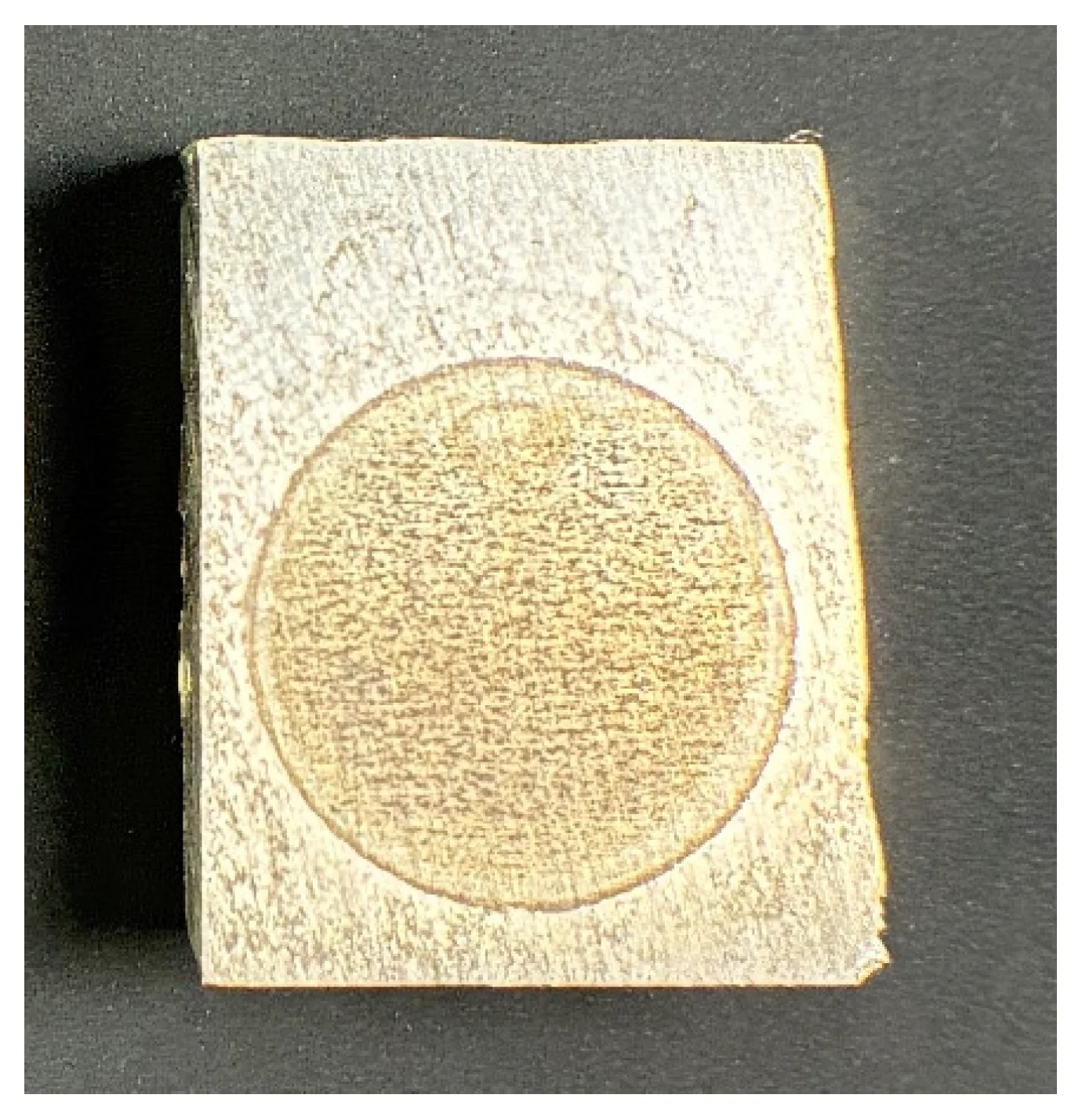
AA2024 verification specimen with a known corrosion area of 143.158 mm^2^ corresponding to the circular corroded region.

The controlled evaluation under standardized conditions (Stage A) was conducted using a corroded AA2024 aluminum alloy specimen with a 143.158 mm^2^ exposure window to H_2_SO_4_. The specimen was used as the working electrode in an electrochemical noise testing cell and exhibited mixed corrosion morphology (Figure 1).

A set of external optical lenses was employed to introduce progressively severe image distortions and evaluate the robustness limits of the proposed methodology by stress testing. The selected lenses generated radial (fisheye), motion-like blur, and kaleidoscopic replication effects, representing extreme acquisition scenarios beyond those typically encountered during normal operation.

Controlled illumination was provided using photographic lighting lamps, enabling consistent lighting conditions throughout the verification procedure. Additionally, a red optical filter was incorporated into the lighting setup for a subset of the images to generate red-illuminated acquisition conditions and evaluate the influence of illumination spectrum on corrosion detection and quantification. Photographs were captured using an iPhone 16e with flash disabled. All images were acquired in PNG format at a resolution of 4284 × 5712 pixels.

**Figure 2:**
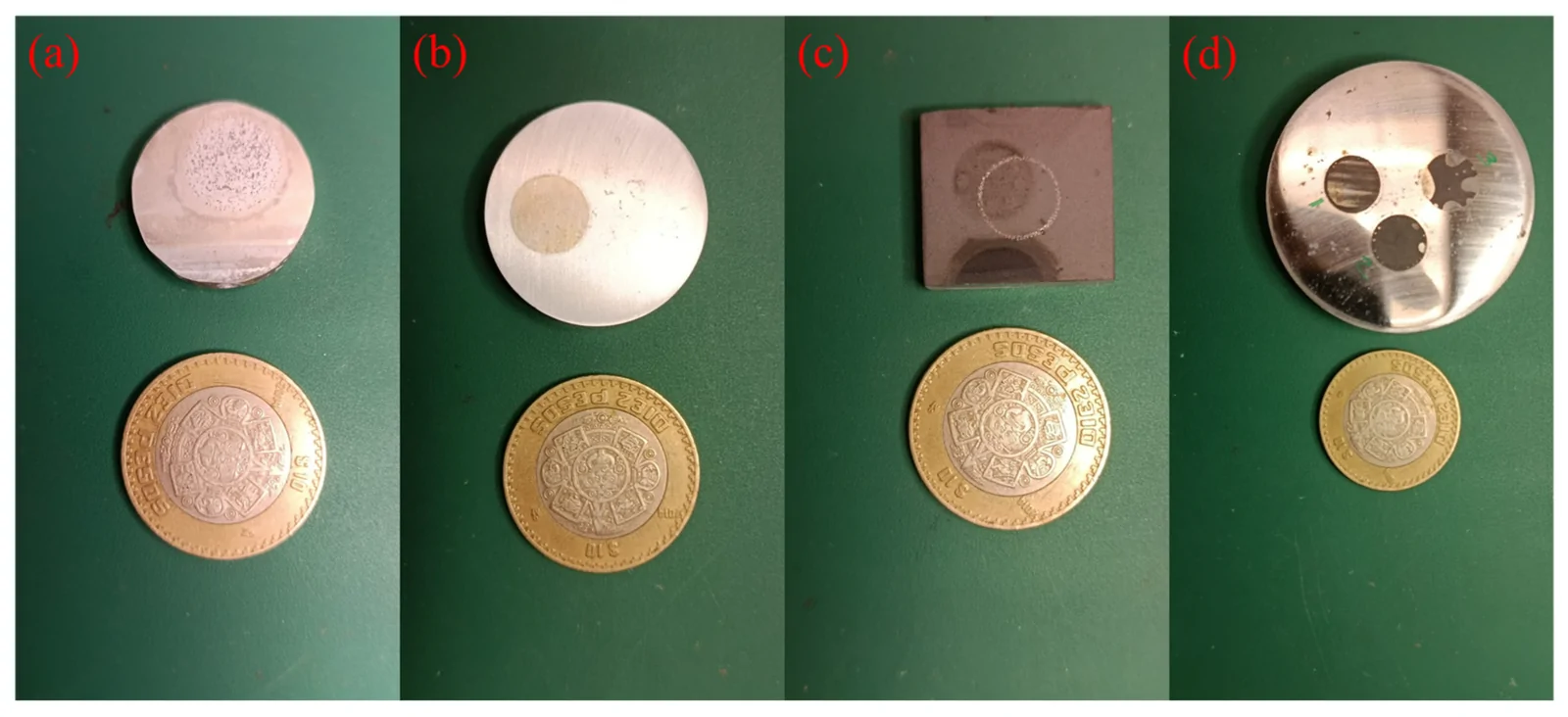
Representative specimens of the multi-specimen database. (**a**) AA2099-1, (**b**) AA2024-2, (**c**) WC-Co-1, and (**d**) AISI 4030-2.

**Figure 3:**
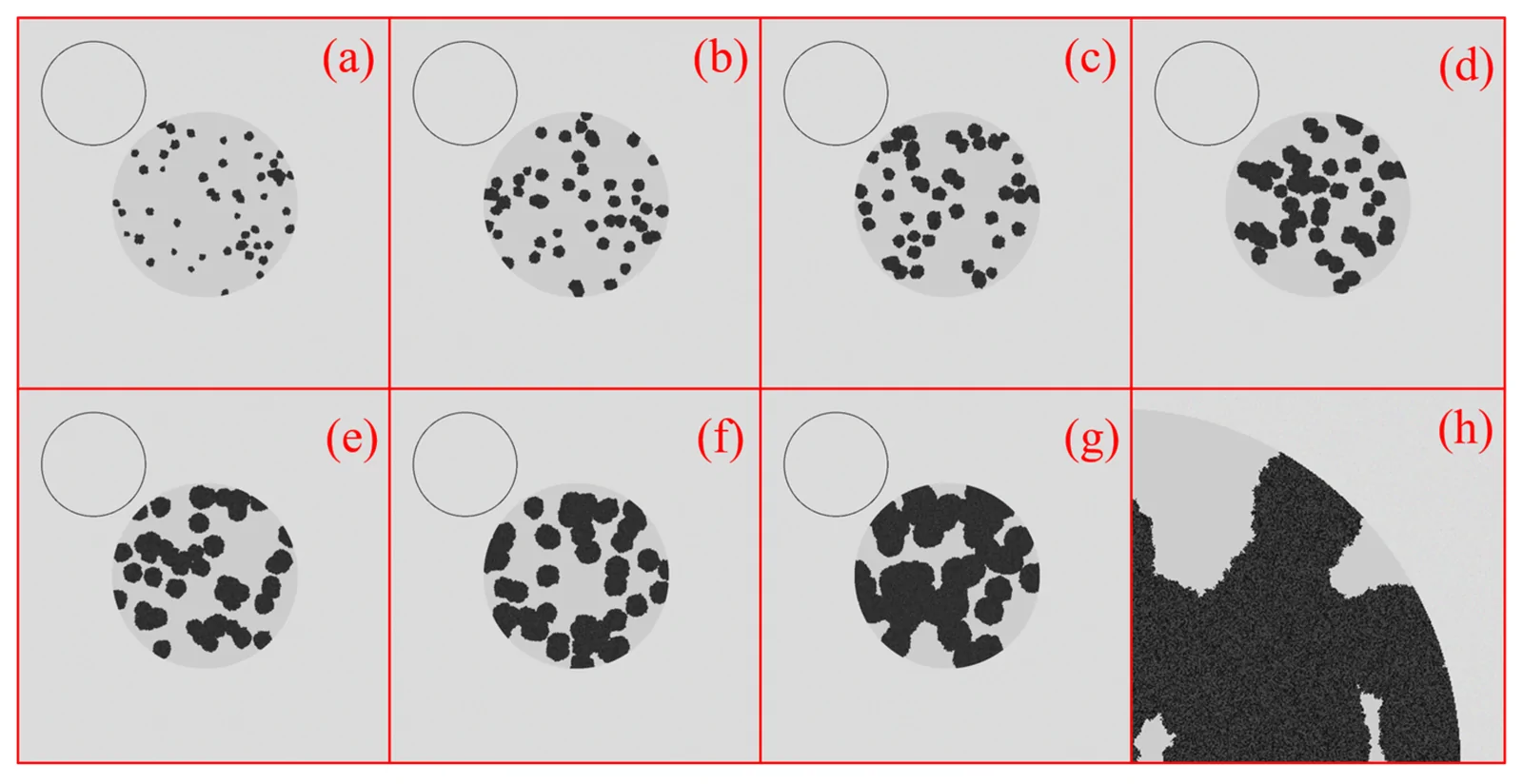
Representative synthetic corrosion coupons generated using a growth probability of 0.90 and 50 initial corrosion nuclei. Target corrosion coverages are (**a**) 10%, (**b**) 20%, (**c**) 25%, (**d**) 35%, (**e**) 40%, (**f**), 50%, and (**g**) 70%, while (**h**) presents a magnified view of the generated corrosion texture.

In stage B, the dataset included aluminum alloys AA2099 and AA2024, AA7075, WC–Co cemented carbide, AISI 4030 steel, and Dual Phase Advanced High Strength Steel (AHSS), (see Figure 2a–d), representing a range of corrosion morphologies and substrate characteristics. Specifically, specimens AA2099-1, AA2024-1, AA7075-1, and Dual Phase AHSS predominantly exhibited localized corrosion (Figure 3a–h). Mixed corrosion morphologies, consisting of localized attack accompanied by regions of more generalized degradation, were observed in specimens AA2024-2, AA2024-3, AA7075-2, AISI 4030-1, and WC–Co-1. Finally, specimens WC–Co-2 and AISI 4030-2 exhibited generalized corrosion, characterized by extensive and relatively homogeneous degradation across the exposed surface. All images were captured under controlled laboratory conditions.

Additionally for Stage C the dataset comprised 42 synthetically generated corrosion coupon images developed to provide controlled reference specimens with known corrosion characteristics. The synthetic dataset incorporated target corrosion coverages of 10%, 20%, 25%, 35%, 40%, 50%, and 70%, together with variations in the number of initial corrosion nuclei (25, 50, and 75) and corrosion growth probabilities (0.75 and 0.90).

A reference coin of 10 Mexican pesos was included in each image to perform dimensional calibration. The resulting corrosion measurements were subsequently compared with the known corrosion area of the specimen and with the reference image to evaluate the consistency of the methodology under different acquisition conditions.

## 3. Results

### 3.1. Controlled Evaluation Under Standardized Conditions

**Figure 4:**
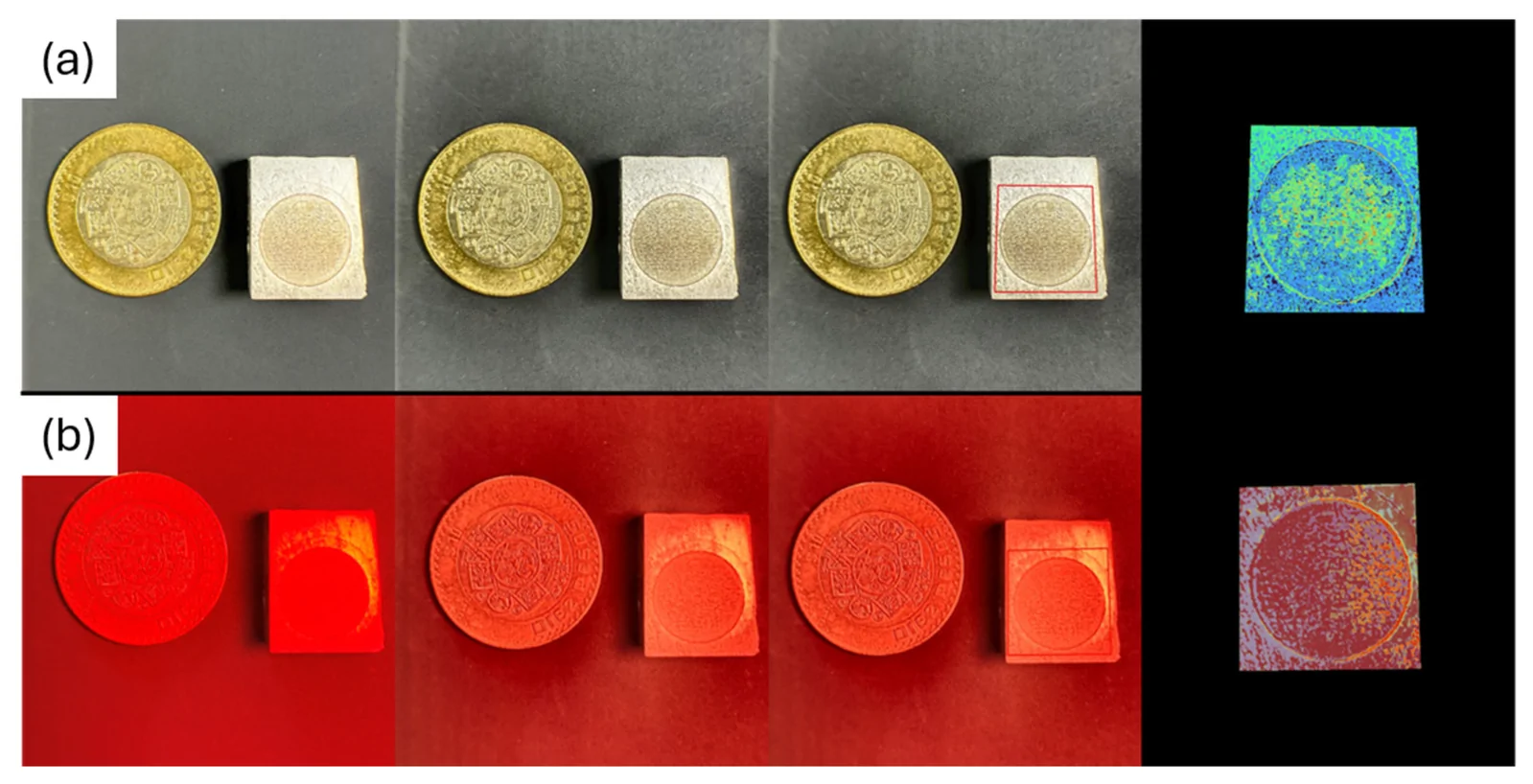
Results obtained under controlled acquisition conditions. (**a**) Standard illumination condition and (**b**) red-illumination condition. From left to right: original image, corrected image, selected region of interest, and resulting corrosion intensity map.

For the reference AA2024 specimen (Figure 4), the proposed methodology estimated a corroded area of 133.64 mm^2^. The specimen contained a circular exposure window of 143.158 mm^2^, corresponding to the physical contact area of the electrochemical cell during testing. This value defines the maximum region exposed to the corrosive environment and was therefore used as a physical reference, resulting in an area-based agreement of 93.35%. However, because the exposed region does not necessarily undergo complete corrosion, an additional segmentation was performed using ImageJ (version 1.54u8; National Institutes of Health, Bethesda, MD, USA) to estimate the localized corroded area. ImageJ measured a corrosion area of 135.97 mm^2^, differing by 2.33 mm^2^ (1.7%) from the value estimated by CorrQuant. In addition, the proposed methodology identified 82 corrosion regions, corresponding to a coverage of 49.75%. The largest detected region occupied 68.1 mm^2^, representing 51% of the total detected corrosion area, while the calculated boundary complexity index was 8.832 (see Table 1).

Under red illumination, the expert-assisted ImageJ segmentation measured a corrosion area of 130.83 mm^2^, while CorrQuant estimated 131.70 mm^2^, corresponding to an absolute difference of 0.87 mm^2^ (0.66%). Relative to the physical exposed area, the CorrQuant measurement corresponded to an area-based agreement of 92.00%. The analysis identified 105 corrosion regions with a coverage of 42.19%. The largest detected region occupied 121.7 mm^2^, accounting for 92.4% of the detected corrosion area, while the calculated boundary complexity index was 3.128.

Comparing the two acquisition conditions, the corrosion area estimated by CorrQuant differed by 1.94 mm^2^, corresponding to a variation of approximately 1.45% relative to the standard illumination measurement. This small difference indicates that the proposed methodology maintains consistent quantitative performance under both standard and red illumination conditions.

#### Distorted Acquisition Conditions

**Figure 5:**
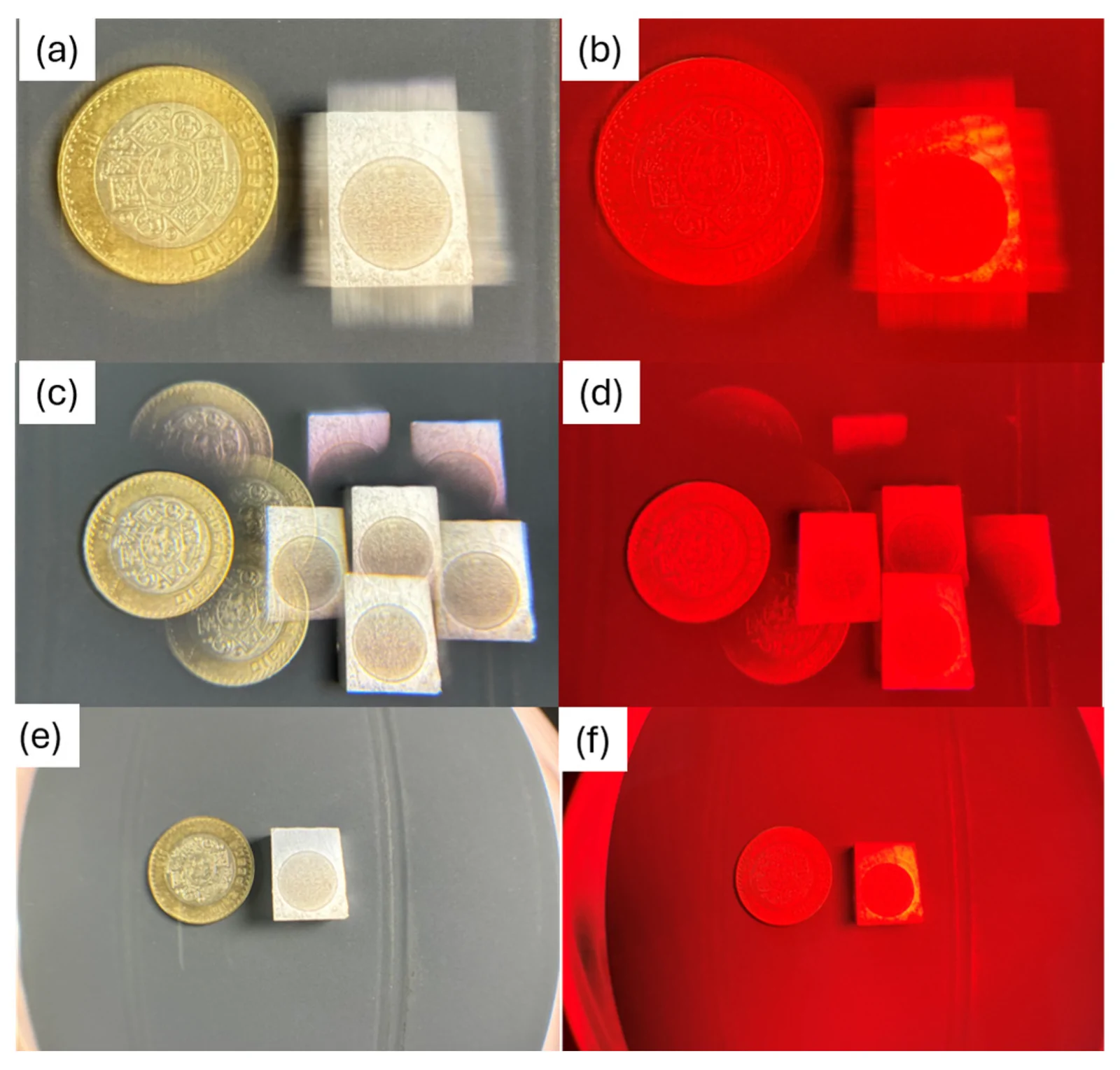
Distorted acquisition conditions used for stress testing. (**a**,**b**) Blur distortion under standard and red illumination, (**c**,**d**) kaleidoscope distortion under standard and red illumination, and (**e**,**f**) fisheye distortion under standard and red illumination. These images represent severe optical distortions introduced to evaluate the operational limits of the proposed methodology.

The corrosion metrics obtained under distorted acquisition conditions are summarized in Table 2. In all cases, the measured corrosion area was lower than the known corrosion area of 143 mm^2^ (Figure 5).

The fisheye images produced measured areas of 87.89 mm^2^ and 113.17 mm^2^ under standard and red illumination, corresponding to accuracies of 61.39% and 79.05%, respectively. The blur images produced measured areas of 105.00 mm^2^ and 93.76 mm^2^, corresponding to accuracies of 73.35% and 65.49%. The kaleidoscope images yielded the lowest measured areas, with values of 66.39 mm^2^ and 91.14 mm^2^, corresponding to accuracies of 46.38% and 63.66%.

The highest area-based agreement within the distorted acquisition conditions was obtained for the fisheye image under red illumination (79.05%), while the lowest area-based agreement was obtained for the kaleidoscope image under standard illumination (46.38%). Substantial variations were also observed in the distribution and morphology metrics, particularly in the number of detected regions, largest region share, and boundary complexity.

### 3.2. Multi-Specimen Analysis

The dataset consisted of 11 images corresponding to AA2024, AA2099, AA7075, WC–Co cemented carbide, AISI 4030, and Dual Phase AHSS, presenting localized attack, mixed corrosion, and generalized corrosion mechanisms. The influence of ROI selection on corrosion area calculations was analyzed by comparing two ROI configurations: a constrained ROI enclosing only the exposed corroded region and a general ROI including the complete sample.

A comparison was established between CorrQuant area measurements and ImageJ area results. ImageJ mask generation was performed using the Threshold tool under both automatic and manual threshold selection, while CorrQuant performed segmentation automatically. During the analysis, ImageJ results were obtained first to avoid bias. Automatic thresholding was performed using the default method, while manual thresholding was performed by comparing the generated mask against the original image and the expected visible corrosion regions.

Table 3 summarizes the corrosion area measurements obtained using ImageJ manual thresholding, ImageJ automatic thresholding, and CorrQuant under constrained and extended ROI configurations for all specimens in addition to ROI sensitivity.

Under the constrained ROI, the three methods generally produced comparable corrosion area measurements, with greater variability observed for ImageJ automatic thresholding in samples 2099-1, 2024-3, 7075-1, 7075-2, and WC–Co-1. Results obtained using ImageJ manual thresholding and CorrQuant showed the greatest resemblance, presenting only minor variations. The greatest discrepancies corresponded to specimens exhibiting localized corrosion (2099-1 and 7075-1) and mixed corrosion (2024-3 and WC–Co-1).

For the general ROI configuration, a similar tenor was observed with some punctual contrasts. ImageJ automatic thresholding produced considerably larger corrosion area measurements than CorrQuant for most specimens. On the other hand, manual thresholding and CorrQuant maintained similar values, with the exception of samples 7075-1 exhibiting localized corrosion, 7075-2 exhibiting mixed corrosion, and WC–Co-2 exhibiting generalized corrosion.

**Figure 6:**
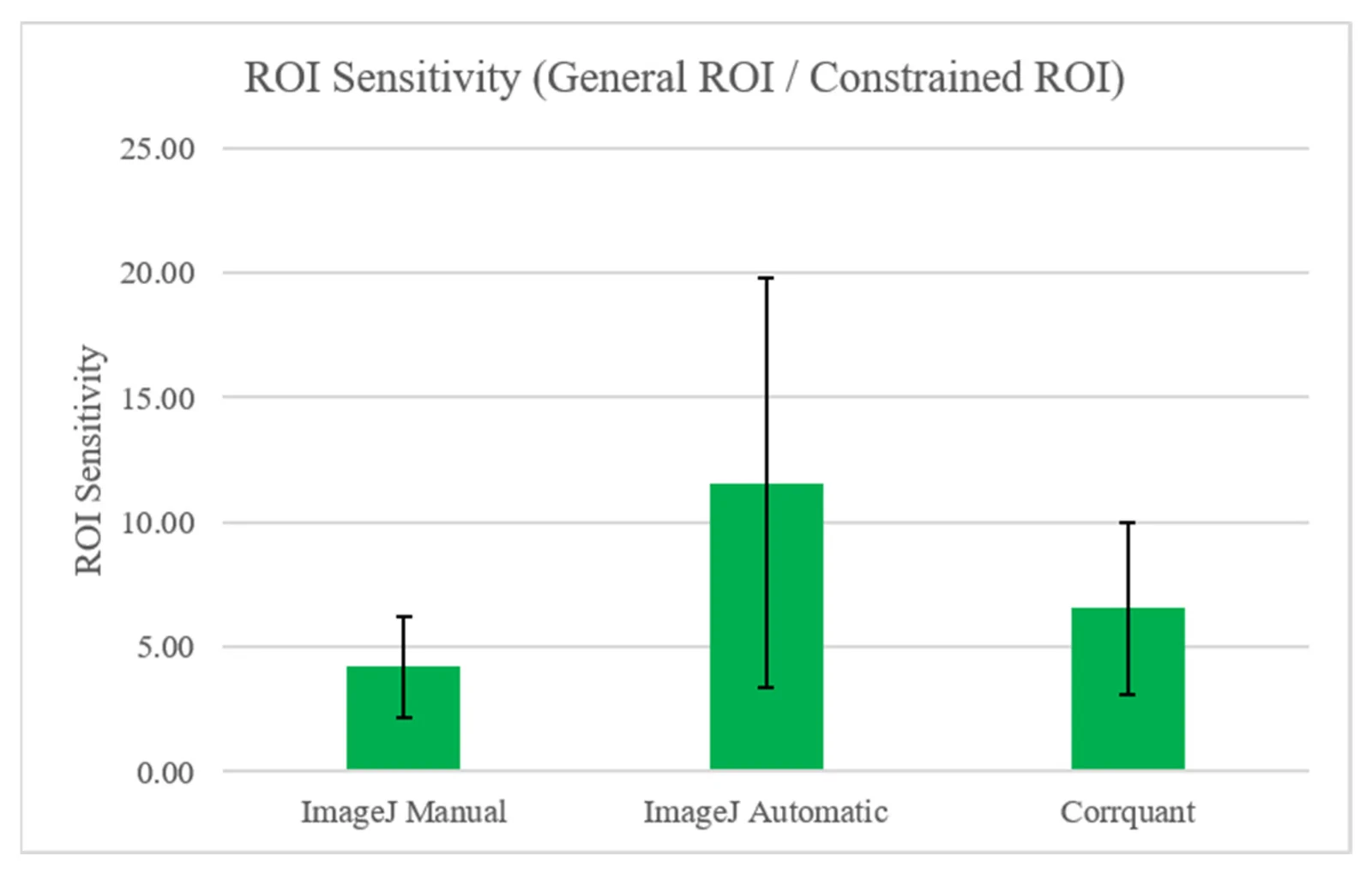
Mean ROI sensitivity (mean ± SD) for each segmentation method.

The observable discrepancy between constrained and general ROI measurements highlights the influence of ROI selection on the reported corrosion area. Since the general ROI incorporates the surrounding material, the measured corrosion area is innately affected by its surface texture, illumination conditions, possible metallic glare, coloration changes, and additional corrosion regions located outside the constrained ROI (Figure 6).

ROI sensitivity was calculated as the ratio between the corrosion area measured under the general ROI and that measured under the constrained ROI. This metric was selected to display the degree to which the calculated corrosion area changes when surrounding surface information is incorporated into the analysis. ImageJ automatic thresholding consistently exhibited the highest ROI sensitivity across all evaluated specimens, whereas manual thresholding produced the lowest values. CorrQuant showed intermediate ROI sensitivity values, remaining substantially closer to manual thresholding than to automatic thresholding.

**Figure 7:**
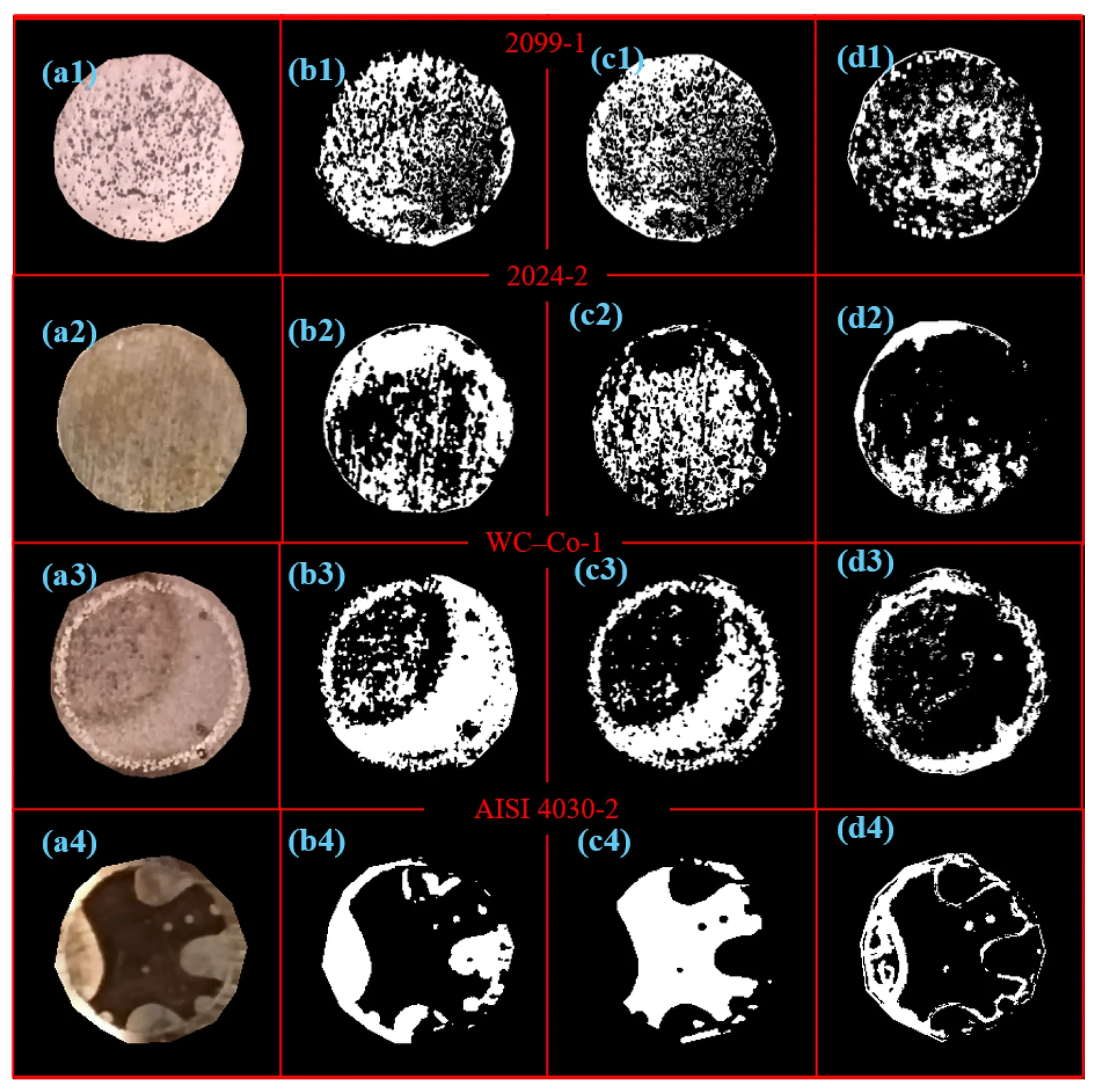
Comparison of corrosion area determination within the constrained region of interest (ROI). (**a1**–**a4**) Original specimen image, (**b1**–**b4**) ImageJ segmentation using automatic thresholding, (**c1**–**c4**) ImageJ segmentation using manually adjusted thresholding, and (**d1**–**d4**) CorrQuant segmentation result.

**Figure 8:**
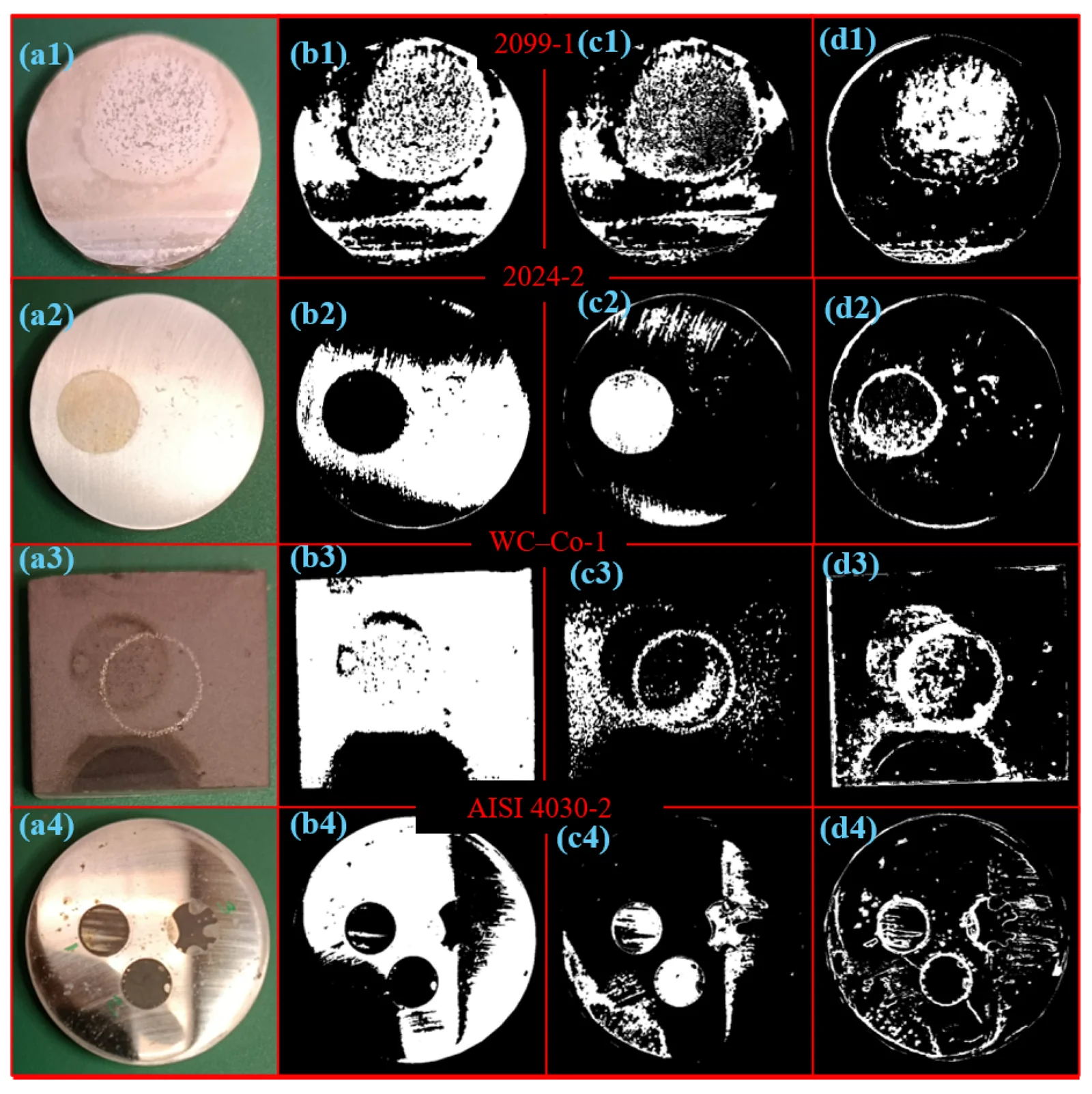
Comparison of corrosion area determination within the general region of interest (ROI). (**a1**–**a4**) Original specimen image, (**b1**–**b4**) ImageJ segmentation using automatic thresholding, (**c1**–**c4**) ImageJ segmentation using manually adjusted thresholding, and (**d1**–**d4**) CorrQuant segmentation result.

Figure 7 and Figure 8 present representative segmentation masks obtained using ImageJ manual thresholding, ImageJ automatic thresholding, and CorrQuant to perform area calculations. Figure 7 (a1–a4,b1–b4,c1–c4,d1–d4) corresponds to the constrained ROI configuration, while Figure 8 (a1–a4,b1–b4,c1–c4,d1–d4) presents its counterpart for general ROI configuration.

### 3.3. Ground-Truth Synthetic Corrosion Evaluation

Corrosion poses a particular challenge in regard to establishing a ground truth reference area from a real-world specimen. The mechanisms present in irregular shapes, sizes, and discolorations, making manual measurements difficult to perform. Due to this, in order to evaluate CorrQuant’s area calculations, synthetic corrosion coupons were developed to establish ground truth. The dataset was developed using Python 3.14.6. with OpenCV and NumPy libraries to generate corrosion patterns. Each synthetic coupon was created on a 100 × 100 mm^2^ virtual canvas at 300 dpi containing a 50 mm circular exposure region and a 28 mm reference coin for dimensional calibration. The resulting synthetic dataset comprised 42 corrosion coupons spanning six corrosion coverage levels, three nuclei configurations, and two growth probabilities.

Corrosion patterns were generated for target surface coverages of 10%, 20%, 25%, 40%, 35%, 50%, and 70% using a stochastic region-growing algorithm. The generation process begins by defining the desired corrosion coverage and the number of corrosion nuclei, which varied between 25, 50, and 75 to produce morphologically distinct corrosion patterns. The number of nuclei controls the spatial distribution of the synthetic corrosion by defining the initial number of independent corrosion colonies, the values of 0.75 and 0.9 were selected. These nuclei were randomly distributed throughout the exposed area to represent independent corrosion initiation sites. Starting from these nuclei, corrosion expanded iteratively through an 8-connected neighborhood, where neighboring pixels were progressively incorporated until the prescribed corrosion coverage was reached. To better approximate real photographic acquisitions, corrosion pixels were assigned randomized grayscale intensities, while low-amplitude Gaussian background noise and Gaussian smoothing (3 × 3 kernel, σ = 0.3) were applied to simulate imaging imperfections and slight optical blur.

The resulting binary corrosion mask was considered the targeted corrosion area. Subsequently, the total affected area was generated by applying a three-iteration morphological dilation to the targeted corrosion mask while constraining the result to the exposed region, the purpose of this operation was to emulate real specimen surface behavior to corrosion with effects such as oxide staining, corrosion products, or different secondary stage corrosion development. Secondary corrosion area was calculated by simply subtracting the targeted corrosion area from the total affected area, representing the surrounding region to principal corrosion. For each generated specimen, the localized corrosion area, affected area, and discoloration area were automatically calculated in mm^2^ using the known image calibration and exported together with their corresponding ground-truth masks for quantitative validation.

The results presented in Table 4 summarize the corrosion area measurement accuracy obtained by comparing the corrosion area estimated by CorrQuant with the known ground-truth corrosion area of each synthetic specimen. Measurement accuracy represents the percentage agreement between both values. To evaluate the operating range of the proposed methodology, the results were grouped according to the target corrosion surface coverage.

Specimens with target coverages of 10% and 20% achieved mean accuracies of 71.22% and 76.32%, respectively. The corresponding mean signed errors were 28.78% and −5.12%, indicating that measurements at low corrosion coverages exhibited greater variability and were susceptible to both over- and underestimation. Although the overall corrosion extent remained relatively small, isolated corrosion regions increased the influence of local segmentation errors on the measured area.

The highest measurement accuracies were obtained for target coverages of 25%, 35%, and 40%, with average accuracies of 91.59%, 93.18%, and 95.56%, respectively. The corresponding mean signed errors of 8.41%, 6.82%, and 4.44% indicate that the proposed methodology produced the most reliable area estimations within this coverage range. These specimens contained sufficiently developed corrosion regions while still maintaining enough contrast between corroded and unaffected areas to support accurate feature extraction and segmentation.

A pronounced reduction in performance was observed for target coverages of 50% and 70%, where average accuracy decreased to 37.78% and 24.54%, respectively. Visual inspection of the segmentation results revealed that, under these conditions, the proposed methodology successfully detected the boundaries of the corrosion regions but failed to segment large homogeneous interior regions. This behavior is attributed to the reduced local contrast within nearly continuous corrosion fields, which limits the effectiveness of the feature-based candidate region generation stage.

To evaluate whether corrosion morphology affected the measurements, results were additionally grouped according to the number of initial nuclei and growth probability. Across all configurations, the majority of specimens (28 of 42) produced overestimations, while the remaining 14 specimens produced underestimations. Overestimated specimens consistently achieved measurement accuracies exceeding 85%, whereas underestimated specimens exhibited substantially lower accuracies, ranging between approximately 31% and 36%. These results suggest that overestimation generally arose from slight expansion of segmented regions, while underestimation was primarily associated with incomplete detection of corrosion interiors in highly homogeneous specimens (Table 5).

## 4. Discussion

Prior to analysis it was anticipated that red illumination would influence the performance of the proposed methodology due to its effect on spectral information. Because the candidate region generation stage incorporates color information through the LAB-b channel, a monochromatic red illumination source was expected to modify the distribution of values within the LAB color space, including those used by the LAB-b voting component, potentially affecting the color-based voting component. However, given that corrosion candidate regions are not identified solely from color information but rather a combination of responses from the LAB-b channel with texture and gradient-based descriptors, the red-illumination condition was introduced to evaluate whether modifications to chromatic information would significantly affect corrosion quantification or whether the remaining voting components would provide sufficient information to maintain detection performance [7,8,12,13,14].

Additionally, the evaluation provided an opportunity to assess the effectiveness of the CLAHE-based image correction stage. Because CLAHE operates by locally enhancing image contrast, it was expected to partially compensate for the reduction in chromatic discrimination introduced by the red illumination condition, thereby preserving the visibility of corrosion-related features used during candidate region generation [13,14].

The optical distortions were selected as stress-test conditions to evaluate the response of the voting framework under acquisition scenarios beyond those typically encountered during routine inspections to explore the operational limits of the system. Blur distortion was expected to reduce local contrast and suppress high-frequency image content, directly affecting the variance, Laplacian, and edge-based voting components. Fisheye distortion was expected to introduce geometric deformation while largely preserving local texture information, potentially affecting region morphology without eliminating the descriptors used for corrosion detection. Kaleidoscope distortion generates multiple artificial replicas of image features and modifies both spatial relationships and local texture patterns, potentially influencing all voting components simultaneously.

The results obtained in the first evaluation stage indicate that the proposed system maintained a consistent estimation of corrosion extent despite substantial changes in illumination spectrum. The measured corrosion areas differed by only 1.94 mm^2^ between the standard and red-illumination conditions, corresponding to a variation of approximately 1.45%. Similarly, both acquisition conditions produced corrosion area-based agreement exceeding 90%, suggesting that the methodology remained capable of identifying the principal corrosion features despite the reduction in available chromatic information [16,19,20,21].

These observations support the design of the candidate region generation stage; Although red illumination was expected to influence the LAB color space distribution, the remaining descriptors continued to provide sufficient information for corrosion identification. The limited reduction in area-based agreement suggests that the voting framework successfully compensated for changes in color information. While corrosion area estimation remained largely unchanged, greater sensitivity was observed in the morphology and distribution metrics. Under red illumination, the number of detected regions increased from 82 to 105, the largest region share increased from 51.0% to 92.4%, and the boundary complexity index decreased from 8.832 to 3.128.

These metrics describe how corrosion pixels are spatially organized rather than the total amount of corrosion detected; The number of detected regions corresponds to the quantity of independent connected corrosion features identified within the analyzed area, while the largest region share represents the proportion of the total detected corrosion area occupied by the dominant corrosion feature. The boundary complexity index quantifies the irregularity of detected corrosion boundaries, with larger values indicating more complex and fragmented morphologies. The limited variation in corrosion area coupled with the substantial changes in these morphology descriptors suggests that red illumination primarily influenced how corrosion pixels were grouped and segmented into individual regions rather than the overall detection of corrosion, appearing to exert a stronger influence on the morphological characterization of corrosion features than on the estimation of total corrosion extent.

Analyzing the distorted acquisition conditions, their intentional design to degrade image information produced the anticipated reduction in corrosion quantification accuracy. Among these conditions, the fisheye images produced the highest accuracies, reaching 61.39% and 79.05% under standard and red illumination, respectively. Although severe radial distortion was visually evident, the underlying texture and edge information remained largely preserved.

This behavior can be attributed to the design of the voting framework. While the fisheye lens altered the global geometry of the image, it did not eliminate the local image descriptors used during candidate region generation. Variance, entropy, Laplacian response, and edge information remained present, allowing corrosion features to continue accumulating votes and be identified as candidate regions. These results suggest that the proposed methodology is more sensitive to the loss of local image information than to geometric deformation alone, provided that the underlying texture and contrast patterns remain recognizable.

Blur condition produced intermediate performance, with area-based agreements of 73.35% and 65.49% under standard and red illumination, respectively. The blur lens degraded local image information and softened feature boundaries through a grid-like diffusion pattern distributed across the field of view. Given the reliance of the voting system on local image descriptors and edge information, this reduction had a severe impact on the results, underestimating the affected area. Nevertheless, the system continued to identify a substantial portion of the corrosion features, suggesting that sufficient texture and contrast information remained available.

The kaleidoscope condition produced the lowest area-based agreement observed during verification, reaching 46.38% and 63.66% under standard and red illumination, respectively. Unlike previous distortions, the kaleidoscope lens fundamentally modifies local image structure by introducing repeated patterns, artificial symmetries, and abrupt changes in texture within the observed specimen, altering multiple descriptor responses simultaneously, making it the most disruptive condition evaluated during verification.

This behavior is reflected in the morphology metrics, particularly the detection of 162 individual regions under standard illumination and the reduction of the largest region’s share to only 18%. While the estimated total corrosion area remained confined to the selected region of interest, the artificial texture patterns introduced by the lens promoted fragmentation of corrosion features into numerous smaller regions. Consequently, the methodology produced a substantial underestimation of corrosion extent. Although such distortions are unlikely to occur during routine inspections, they provide valuable insight into the operational limits and influences on the system.

Taken together, Stage A analysis demonstrated that the proposed methodology is more sensitive to distortions affecting spatial and structural image information than to changes in illumination spectrum. Under controlled acquisition conditions, corrosion area estimation remained above 90% area-based agreement despite substantial modifications in color information. In contrast, severe optical distortions reduced area-based agreement by degrading the texture, contrast, and geometric characteristics utilized during candidate region generation. Nevertheless, measurable corrosion quantification was maintained across all evaluated conditions, supporting the multi-descriptor voting design and identifying the acquisition scenarios most likely to affect system performance.

Pertaining to the multi-specimen analysis, the selection of samples with various corrosion mechanisms and ROI selection conditions were chosen to determine CorrQuant’s sensitivity to detect localized, generalized, and mixed corrosion when sample ROI area was constrained, as well as the effects of sample background information to isolate areas of interest against accompanying textures, metallic surface conditions, and additional corrosion areas to the constrained ROI.

The area results for the constraint ROI showed greater resemblance to ImageJ manual thresholding, this is due to the human intervention when selecting the mask by user supervision against the original image to isolate the intended areas of interest. Considering the autonomous operation of CorrQuant from the user, the automatic thresholding of ImageJ would be more comparable and highlights CorrQuant’s ability to isolate corrosion areas more succinctly, supported by the manual thresholding results.

Regarding the area results of general ROI selection, greater discrepancies were found amongst the three methodologies, maintaining the greatest similarities between manual thresholding and CorrQuant. This yawning difference is mainly attributed to the individual design of operations from each methodology, where it was found that automatic thresholding has a proneness to detect the negative areas, selecting the unaffected zones rather than corrosion; for both ImageJ methodologies it was also observed that significant textural and lighting conditions interfered with corrosion area isolation. In CorrQuant’s case it was observed that neither negative space nor lighting surface conditions affected its capacity to detect corrosion. However, it was noted that under certain corrosion mechanisms there was only partial area identification. This will be expanded upon further in the following paragraphs on specific mechanisms.

Viewed in its entirety, the comparison of ROI conditions demonstrates that for CorrQuant’s ability to detect the area of corrosion mechanisms it is ideal to isolate the region of interest as closely as possible. Additionally, it demonstrated that it remained unaffected by most surface features regarding lighting, glare, and reflection, which are unavoidable characteristics of metallic materials.

It was therefore described that the magnitude of influence of background has a significant impact on final calculations as seen by the ROI Sensitivity measurements. Manual thresholding showed the lowest ROI sensitivity because the operator was able to visually exclude surrounding surface texture when selecting the corrosion regions. In contrast ImageJ automatic thresholding frequently incorporated non-corroded regions with similar intensity characteristics, resulting in considerably larger increases in measured area when the ROI was expanded. CorrQuant exhibited lower ROI sensitivity than ImageJ automatic thresholding across most specimens, suggesting that the proposed segmentation strategy is less affected by the inclusion of surrounding material. This behavior is likely associated with the combination of multiple feature maps and subsequent region-growing constraints, which reduce the influence of isolated texture variations compared with intensity-based thresholding alone.

Concentrating now on the specific corrosion mechanisms and their results:

For localized corrosion under constrained ROI: (2099-1, 2024-1, 7075-1, Dual Phase AHSS): ImageJ automatic thresholding identified the primary corroded regions in sample 2099-1. The remaining samples exhibited inverse segmentation by detecting the uncorroded regions instead of the corrosion areas. Manual thresholding for first three samples successfully identified the corrosion areas, while Dual Phase AHSS maintained same issue under automatic thresholding. CorrQuant successfully identified the primary corrosion regions in all localized corrosion specimens, with a slight over-segmentation in sample 7075-1 caused by a discoloration region being interpreted as corrosion.

For localized corrosion under general ROI (2099-1, 2024-1, 7075-1, Dual Phase AHSS): ImageJ automatic thresholding incorrectly incorporated background information into the corrosion area for all samples. Manual thresholding considerably reduced this effect, particularly for samples 2099-1, 2024-1, and 7075-1, although Dual Phase AHSS specimen still presented significant glare being identified as corrosion. CorrQuant provided the greatest isolation of the principal corrosion regions while still detecting additional corrosion instances located outside the constrained ROI mostly unaffected by illumination artifacts. Sample 7075-1 presented greater background selection due to the pronounced surface texture of the specimen, which was expected.

For generalized corrosion under constrained ROI (WC-Co-2, AISI 4030-2): both specimens exhibited generalized corrosion with portions of the surface remaining partially or completely unaffected. For ImageJ automatic thresholding presented inverse segmentation of the affected areas in both cases. Manual thresholding improved this behavior; sample WC-Co-2 successfully identified the corrosion regions, although with over-segmentation that included partially unaffected areas. Sample AISI 4030-2 presented the closest agreement with the visually identified corrosion region. CorrQuant partially identified the corrosion area in WC-Co-2, underestimating the generalized corrosion region. For sample AISI 4030-2, only the corrosion boundaries and portions of the affected regions were identified.

For generalized corrosion under general ROI (WC-Co-2, AISI 4030-2): Both samples presented additional corrosion areas besides the one selected for constrained ROI. All methodologies generally maintained the same segmentation behavior observed under the constrained ROI while additionally identifying the newly incorporated corrosion regions. Surface texture affected all three methodologies; however, illumination conditions and metallic glare substantially influenced both ImageJ implementations, particularly automatic thresholding. CorrQuant remained largely insensitive to illumination effects, with segmentation differences arising primarily from surface texture and minor discoloration regions.

For mixed corrosion under constrained ROI (2024-2, 2024-3, 7075-2, AISI4030-1, WC-Co-1): ImageJ automatic thresholding presented over-estimation and inverse segmentation selection in first three samples. In the case of WC-Co-1 the main corrosion area was identified, yet discoloration areas were also incorrectly classified as corrosion. Manual thresholding successfully identified the corrosion regions in the first three specimens, while WC-Co-1 maintained the same limitation observed under automatic thresholding. CorrQuant correctly identified the localized corrosion components in samples 2024-2 and WC-Co-1 but underestimated the generalized corrosion regions. The remaining specimens successfully represented the corrosion morphology, where the localized components constituted the majority of the affected surface.

For mixed corrosion under general ROI (2024-2, 2024-3, 7075-2, AISI4030-1, WC-Co-1): ImageJ automatic thresholding exhibited inverse segmentation in all samples. Manual thresholding presented an improvement in samples 2024-2 and 2024-3 with some texture from background being selected. Samples 7072-2 presented a significant detection of the illumination gradient on the metallic surface as corrosion. Sample WC-Co-1 successfully identified the principal corrosion region, however it also presented inverse segmentation of portions of the generalized corrosion component. CorrQuant presented the same behavior observed under the constrained ROI for sample 2024-2 with partial recognition of all corrosion components with minimal background influence. Sample 2024-3 presented the closest agreement among the three methodologies, with minimal interaction with the surrounding background. Sample 7075-2 did not exhibit the illumination artifacts present in both ImageJ iterations, successfully isolating the principal corrosion region while additionally identifying corrosion instances outside the constrained ROI with minimal background interactions. Sample WC-Co-1 presented the closest overall agreement among the evaluated methodologies; however, additional generalized components present in the sample outside the constrained ROI were not identified.

These observations emphasize that CorrQuant possesses a greater ability to detect localized corrosion while reducing interference from illumination conditions, metallic glare, and surface discoloration compared to conventional thresholding approaches. Generalized corrosion, however, tends to be underestimated by the proposed methodology. This behavior is associated with the candidate region generation strategy employed by CorrQuant. The proposed methodology identifies corrosion candidates by combining multiple complementary image descriptors through a voting-based consensus before refining the resulting regions using morphological operations and similarity-constrained region growing. This behavior represents an inherent trade-off of the proposed methodology. CorrQuant was intentionally designed to require consistent evidence across the multiple complementary image descriptors before classifying a region as corrosion. This conservative candidate generation strategy effectively suppresses isolated responses from individual descriptors, thereby reducing false-positive detections. However, the same strategy also limits sensitivity to generalized corrosion, where large homogeneous regions often provide weaker consensus across the selected descriptors. Consequently, generalized corrosion tends to be conservatively segmented. Recognizing this crossroads, future work will seek to improve the detection of generalized corrosion while maintaining the robustness of the proposed methodology.

Additionally, needful supplementary segmentation evaluation metrics will be incorporated as subsequent developments of the proposed methodology are undertaken to further strengthen the validation of the proposed methodology and establish a more comprehensive performance assessment. Currently, comparison against the ImageJ methodology provides a practical reference for both qualitative segmentation behavior and quantitative corrosion area measurements. However, since the ImageJ thresholding approach itself does not constitute a pixel-level ground-truth reference, it cannot fully replace standardized segmentation evaluation protocols based on annotated reference masks. Therefore, the present validation should be interpreted as a comparative evaluation against a conventional image-processing methodology, and this limitation should be considered when assessing the reported segmentation performance.

Observing Figure 5, Sample 2099-1, corresponding to localized corrosion, the (b) and (c) masks, corresponding to the ImageJ automatic and manual thresholding methodologies, respectively, show significant detection of the sample’s non-corroded surface as part of the corrosion area, most notably the crescent-like shape at the bottom of the specimen. In turn, image (d) detects the localized corrosion regions with greater detail while preserving the visible morphology of the attack, albeit appearing slightly larger than the original features due to the conservative refinement strategy employed by CorrQuant.

Sample 2024-2, corresponding to mixed corrosion, presents in (a) the original ROI of the sample, exhibiting distinct localized attack points in the lower portion, although faint, together with a faint cloud-like component in the center-right and top-left regions, coinciding with the initiation of generalized corrosion on the compromised surface. The discoloration is subtle but visually distinguishable. Images (b) and (c) identify the lighter-toned discoloration at the top of the sample as corrosion, while additionally classifying a large portion of the remaining surface as corroded, likely influenced by the vertically oriented surface scratches. In contrast, image (d) preserves finer detail within the principal localized corrosion regions, particularly the attacks located in the lower-center and lower-right portions of the specimen. Although slight overselection of neighboring regions is still observed, the segmentation more closely resembles the visible localized corrosion morphology. The faint generalized discoloration, however, is not fully detected.

Sample WC-Co-1 presents a particular mixed corrosion case consisting of a localized concentric ring, within which a partial imprint of a second localized attack is observed together with surrounding generalized corrosion regions. In both images (b) and (c), the unaffected crescent-shaped region inside the concentric attack is incorrectly classified as corrosion, while the additional attack within the ring is only partially detected through isolated localized regions. In contrast, image (d) preserves the concentric attack while distinguishing two separate localized attacks inside the ring. The surrounding corrosion mechanism is also detected; however, only its most distinctive localized regions are identified rather than the complete homogeneous area, resulting in a segmentation that more closely follows the visible corrosion morphology.

Sample AISI 4030-2 showcased the principal limitation of the proposed methodology. This specimen possesses an irregular generalized attack resembling the letter “K”, where the corroded surface is highly homogeneous with minimal discoloration throughout the affected region. Image (c) captures most of the generalized corrosion area, whereas image (d) produced by CorrQuant primarily detects the outline of the corrosion pattern. This observation emphasizes the aforementioned limitation associated with generalized corrosion, where highly homogeneous corrosion surfaces provide insufficient distinctive features for the conservative candidate generation strategy, resulting in partial segmentation of the affected region.

Figure 6, presenting the generalized ROI, Sample 2099-1 exhibits localized attack at the top center of the specimen surrounded by corrosion by-products. At the bottom of the sample, noticeable discoloration is visible due to the anodizing process performed prior to corrosion testing. Images (b) and (c) capture considerable detail of both the localized attack and the surrounding corrosion by-products. However, the unaffected lower region of the specimen is also identified as corrosion, likely influenced by the anodizing discoloration. In contrast, image (d) detects the principal localized attack with slightly less detail, while significantly reducing the detection of corrosion by-products and preserving the remaining non-corroded surface. Only a few features associated with the anodized region at the bottom are identified.

Sample 2024-1 presents mixed corrosion, consisting of a well-defined circular corrosion region together with additional localized attacks distributed throughout a surface exhibiting fine scoring marks. In both images (b) and (c), the background texture considerably influences the segmented area, with the principal corrosion region being detected almost in its entirety but accompanied by substantial detection of the surrounding surface. In contrast, image (d) preserves the circular outline while distinguishing the individual localized attacks within it. Compared to the constrained ROI, the segmentation maintains a similar morphology, unlike the ImageJ methodologies. Additionally, several of the faint localized attacks surrounding the principal region are also detected.

Sample WC-Co-1, consisting of a matte metallic surface exhibiting multiple corrosion mechanisms, presents a principal concentric localized attack containing a secondary offset mixed corrosion region. At the bottom of the specimen, a semicircular generalized corrosion region with corrosion by-products is also present. Image (b) produces an unusual segmentation, classifying nearly the entire ROI as corrosion while failing to identify most of the generalized attack at the bottom and portions of the mixed corrosion region. In image (c), the concentric ring is preserved; however, background texture is also detected, while only a limited portion of the mixed corrosion region is identified. The generalized corrosion region is largely absent. In contrast, image (d) also preserves the concentric attack, although with slightly less detail than image (c), while additionally detecting the mixed corrosion region inside the ring. The generalized corrosion at the bottom is faintly outlined together with portions of the surrounding corrosion by-products, providing the closest visual resemblance to the observed corrosion morphology.

Sample AISI 4030-1 presents three distinct generalized corrosion regions with different morphologies: one consisting of partial horizontal-line attack, a second where nearly the entire circular area has been consumed, and a third resembling the letter “K”. The specimen possesses a mirror-like surface that produces strong reflections on the left-hand side together with a vertical discoloration band extending from top to bottom on the right. Image (b) captures the general corrosion outlines; however, the segmentation is largely inverted while additionally classifying substantial regions associated with illumination and surface discoloration as corrosion. Image (c) provides the closest representation of the principal generalized corrosion regions, although it remains affected by the metallic surface characteristics. Finally, image (d) clearly preserves the outlines of the principal corrosion regions together with several additional localized corrosion sites while exhibiting the least interference from the metallic surface characteristics. However, the interior of the generalized corrosion regions is not fully detected, reiterating the previously discussed limitation associated with highly homogeneous corrosion surfaces.

With regard to the ground-truth synthetic corrosion evaluation, first, the generation of corrosion coupons accounted for real life surface variabilities such as random corrosion initiation sites, irregular corrosion growth patterns, grayscale intensity variations, and imaging noise. The reported results revealed several important characteristics. First, specimens with corrosion coverages below 20% were susceptible to measurement variability, as the limited extent of the corrosion regions increased the influence of surrounding background information and led primarily to overestimation. As the target corrosion coverage increased to between 25% and 40%, the methodology consistently achieved its highest measurement accuracies, ranging from 91 to 95%, indicating that localized corrosion events with well-defined boundaries are reliably detected and quantified by the proposed system. Within this operating range, the mean signed error remained low, varying between +4.44% and +8.41%, reflecting minimal systematic measurement bias.

Interestingly, variations in the number of corrosion initiation nuclei and growth probability produced comparatively small differences in measurement accuracy, suggesting that the quantitative performance of CorrQuant is influenced more by the overall corrosion coverage than by moderate changes in the spatial distribution of localized corrosion. The highest individual result was obtained for the specimen with a target coverage of 40%, 75 initial nuclei, and a growth probability of 0.75, achieving a measurement accuracy of 99.25% relative to the ground-truth corrosion area. This result indicates that CorrQuant is capable of producing highly accurate corrosion area estimations when the corrosion morphology falls within its intended operating conditions.

On the other hand, the operational limits of the proposed methodology became evident for specimens with corrosion coverages between 50% and 70%. This observation is consistent with the previous analysis of homogeneous corrosion regions, where the system exhibited a tendency to identify primarily the boundaries of the corrosion, excluding the interior from the corrosion area. This limitation is further reflected by the pronounced negative mean signed errors of −62.22% and −75.46% for the 50% and 70% coverage levels, respectively, demonstrating a systematic tendency to underestimate corrosion area as the surface becomes increasingly homogeneous. The results suggest that the limiting factor is not the complexity or distribution of localized corrosion, but rather the progressive loss of local image contrast as corrosion becomes increasingly homogeneous, reducing the discriminative capability of the current feature extraction and candidate region generation strategy. These findings highlight two important conclusions. First, CorrQuant provides reliable quantitative measurements for localized corrosion, which represents the primary objective of the proposed methodology. Second, extensive or generalized corrosion requires additional refinement of the segmentation pipeline before comparable quantitative performance can be achieved.

## 5. Conclusions

- This work presented CorrQuant, a web-based corrosion quantification platform designed to estimate geometrical and morphological descriptors of surface corrosion through a computer vision pipeline integrating geometric calibration, image enhancement, feature extraction, adaptive region identification, distribution analysis, morphological characterization, and quantitative assessment. The methodology employed by the platform combines chromatic, textural, and structural descriptors through a consensus-based voting strategy to identify corrosion regions and provide quantitative estimations of corrosion area, coverage percentage, detected region count, largest region area, largest region share, and boundary complexity, as well as corrosion intensity maps.
- Analysis using a specimen with known corrosion extent demonstrated corrosion area estimation with area-based agreement exceeding 90% under controlled acquisition conditions. Evaluation under optical stress-test conditions further showed that the methodology is considerably more sensitive to distortions affecting local image information rather than variations in illumination spectrum, establishing the current operational limits of the proposed approach. The values reported by CorrQuant should be interpreted as quantitative estimations rather than absolute measurements.
- Experimental evaluation across multiple metallic materials exhibiting localized, mixed, and generalized corrosion demonstrated that CorrQuant consistently quantifies localized corrosion while identifying the current limitations of the methodology for increasingly homogeneous corrosion surfaces. Comparison against ImageJ highlighted the advantages of the proposed methodology for corrosion quantification, providing automated and reproducible measurements while avoiding the variability associated with manual threshold selection.
- Synthetic coupons for ground-truth validation demonstrated that CorrQuant performs most reliably for localized corrosion covering approximately 25–40% of the exposed surface, achieving measurement accuracies between 91% and 96%, while performance decreases as corrosion transitions toward generalized, homogeneous attack due to reduced local image contrast.
- Beyond the numerical results, this work demonstrates the feasibility of transforming visual corrosion observations into quantitative information through an accessible web-based platform. By performing computationally intensive image processing on the hosting server, CorrQuant reduces the technical, computational, and economic barriers associated with image-based corrosion assessment without requiring specialized equipment or programming knowledge.
- Future work will focus on expanding the verification database, refining the multi-descriptor voting framework, incorporating benchmark datasets with expert-generated pixel-level annotations to complement the present validation strategy, and extending the platform toward automated morphology classification and corrosion mechanism identification. These developments will require larger validated datasets capable of relating observed corrosion morphology to confirmed corrosion mechanisms.
- As CorrQuant continues its development, the platform remains available in beta form for testing and evaluation. Researchers, students, inspectors, and industry professionals are encouraged to utilize the system and provide feedback on its performance. User-submitted results, observations, and recommendations will contribute to future improvements and help guide the continued development of image-based corrosion quantification methodologies.

## Figures and Tables

**Table 1:** Standard conditions results.

Standard Reference Sample		Standard Reference Sample Red Lighting	
Area CorrQuant (mm^2^)	133.64	Area (mm^2^)	131.7
Area ImageJ (mm^2^)	135.97	Area ImageJ (mm^2^)	130.83
Area-based agreement	93.35%	Area-based agreement	91.99%
Coverage	49.75%	Coverage	42.19%
Detected regions	82	Detected regions	105
Largest Region (mm^2^)	68.1	Largest Region (mm^2^)	121.7
Largest Region Share	51%	Largest Region Share	92.4%

**Table 2:** Distorted conditions results.

Blur Sample		Blur Sample Red Lighting	
Area (mm^2^)	105	Area (mm^2^)	93.76
Area-based agreement	73.34%	Area-based agreement	65.49%
Coverage	35.30%	Coverage	32.18%
Detected regions	70	Detected regions	43
Largest Region (mm^2^)	82.73	Largest Region (mm^2^)	75.72
Largest Region Share	78%	Largest Region Share	80.8%
Boundary complexity	2.623	Boundary complexity	4.844
Kaleidoscope sample		Kaleidoscope sample red lighting	
Area (mm^2^)	66.39	Area (mm^2^)	91.14
Area-based agreement	46.37%	Area-based agreement	63.66%
Coverage	25.47%	Coverage	29.67%
Detected regions	162	Detected regions	64
Largest Region (mm^2^)	11.97	Largest Region (mm^2^)	45.72
Largest Region Share	18%	Largest Region Share	50.2%
Boundary complexity	4.18	Boundary complexity	4.046
Fisheye sample		Fisheye sample red lighting	
Area (mm^2^)	87.89	Area (mm^2^)	113.17
Area-based agreement	61.39370486	Area-based agreement	79.0525154
Coverage	26.06%	Coverage	35.15%
Detected regions	46	Detected regions	56
Largest Region (mm^2^)	42.2	Largest Region (mm^2^)	74.95
Largest Region Share	48%	Largest Region Share	66.2
Boundary complexity	6.456	Boundary complexity	4.156

**Table 3:** Corrosion area measurements and ROI sensitivity values.

Specimen	Constraint ROI			General ROI			ROI Sensitivity		
	Manual Thresholding	Automatic Thresholding	CorrQuant	Manual Thresholding	Automatic Thresholding	CorrQuant	Manual Thresholding	Automatic Thresholding	CorrQuant
2099-1	70.04	105.81	75.43	171.77	281.42	163.68	2.45	2.66	2.17
2024-1	64.94	70.25	66.28	184.66	845.00	187.73	2.84	12.03	2.83
2024-2	43.81	51.46	33.53	158.80	410.43	151.46	3.62	7.98	4.52
2024-3	65.49	107.03	66.96	171.42	846.11	178.23	2.62	7.91	2.66
7075-1	66.54	93.79	66.76	358.41	785.44	644.57	5.39	8.37	9.66
7075-2	30.79	61.57	27.16	69.18	404.85	185.21	2.25	6.58	6.82
WC–Co-1	47.40	69.37	45.23	96.09	510.17	214.56	2.03	7.35	4.74
WC–Co-2	40.99	21.84	37.70	263.33	502.08	341.37	6.42	22.99	9.05
AISI 4030	68.76	74.66	66.76	538.10	873.66	496.78	7.83	11.70	7.44
AISI 4030	66.52	43.95	36.90	417.70	1364.96	472.94	6.28	31.05	12.82
Dual Phase AHSS	129.07	130.88	75.28	581.33	1138.72	701.18	4.50	8.70	9.31

**Table 4:** Mean measurement accuracy and signed error as a function of target corrosion coverage for the synthetic validation dataset.

Target Coverage (%)	Mean Accuracy (%)	Mean Signed Error (%)
10	71.22	28.78
20	76.32	−5.12
25	91.59	8.41
35	93.18	6.82
40	95.56	4.44
50	37.78	−62.22
70	24.54	−75.46

**Table 5:** Quantitative performance as a function of the number of initial corrosion nuclei.

Initial Corrosion Nuclei	Mean Accuracy (%)	Mean Overestimation Accuracy (%)	Mean Underestimation Accuracy (%)
25	65.87	89.10	34.90
50	72.34	88.49	31.94
70	71.87	86.20	36.06

## Data Availability

The original contributions presented in this study are included in the article. Further inquiries can be directed to the corresponding authors.
